# Impact of COVID-19 on the severity and length of stay among hospitalized pediatric patients

**DOI:** 10.3389/fped.2026.1849661

**Published:** 2026-08-31

**Authors:** Faria Abdullah, Marion Hare, Elizabeth Tolley, Jason Yaun

**Affiliations:** 1 General Pediatrics, Le Bonheur Children’s Hospital, Memphis, TN, United States; 2 Department of Pediatrics, University of Tennessee Health Science Center, Memphis, TN, United States; 3 Department of Preventive Medicine, University of Tennessee Health Science Center, Memphis, TN, United States

**Keywords:** COVID-19, emergency room visits, intensive care units, patient acuity, pediatric hospitals

## Abstract

**Objective:**

Pediatric hospital admissions and emergency department visits decreased during the COVID-19 pandemic. The primary objective of this study was to determine if pediatric patients hospitalized during the pandemic were more severely ill compared with those during the pre-pandemic period by measuring severity of illness, intensive care unit admissions, and length of stay.

**Methods:**

A cross-sectional study was conducted of patients admitted to an urban, large children's hospital in the Mid-Southern US from 2016 to 2021. A total of 58,369 admissions were included, with 42,877 pre-pandemic and 15,492 pandemic admissions.

**Results:**

During the pandemic, admissions of more severe illnesses increased significantly compared with pre-pandemic admissions. Intensive care unit admission rates increased during the pandemic (11%) compared with pre-pandemic levels (9.9%). Length of stay did not differ.

**Conclusion:**

The severity of illness for hospitalized pediatric patients increased significantly during the COVID-19 pandemic. The increase may stem from unmet medical needs; patients must still receive routine, acute, and chronic care.

## 1 Introduction

On March 11, 2020, the World Health Organization (WHO) officially declared COVID-19 a pandemic (1). In response, governments and business entities placed restrictions on daily activities, affecting all facets of life, including travel, work, leisure activities, and access to routine medical care. From March 19 to April 2, 41 states issued “Stay-at-Home” orders (2). By late March and early April, more than 310 million Americans were under directives to shelter in place (3). Most major events worldwide were canceled or postponed. The rationale behind the national lockdown was that social distancing could impede the spread of the disease.

The pandemic was marked by different strains of the COVID-19 virus that emerged and affected different populations in a variety of ways. For children, the initial wild-type and Alpha variants did not produce notable numbers of infections or hospitalizations due to lockdowns, protective factors, and the use of virtual platforms for education. Studies showed significantly lower numbers of pediatric outpatient visits, emergency room visits (4), and hospitalizations during this time (5). Pediatric hospital beds were even used for adult patients in areas with overwhelming numbers of adult COVID-19 hospitalizations (6).

The emergence of the Delta and Omicron variants had a greater detrimental effect on the number of pediatric cases and hospitalizations. The initial Delta variant outbreak occurred in April 2021 in India and rapidly spread throughout the world, causing a surge in pediatric hospitalizations in the US (7). In November 2021, the highly contagious Omicron variant emerged (8), and subsequently caused the largest surge of cases and hospitalizations to date.

During the COVID-19 pandemic, children continued to require routine medical care, ongoing chronic care management, and emergency department care for injuries and other illnesses. Given the decreased access to care, shift of resources to adult patients, and general avoidance of healthcare facilities during this time, pediatricians became concerned about children's access to care and immunizations, and the potential exacerbating effects of access for chronic illnesses and chronic conditions. There were also uncertainties about delayed presentations for acute injuries, mental health conditions, and other illnesses that could require hospital admission. Together, these conditions alarmed pediatricians, raising concern that children who were admitted to the hospital may have presented with higher acuities, which could require higher levels of care and increased rates of intensive care unit (ICU) admissions, and longer lengths of stay due to limited access to care, avoidance of healthcare systems, or other factors that may have led to later presentations with more severe illnesses than what would have otherwise happened in the absence of the pandemic.

The purpose of the study was to compare overall pediatric hospitalization patterns during pre-pandemic and pandemic periods by determining the following: (1) did pediatric patients admitted to a tertiary care children's hospital in the Mid-Southern US, during the pandemic period have higher severity levels than those admitted during the pre-pandemic period; (2) was there a difference between the rates of ICU admissions in these cohorts; and (3) was there a difference in the average lengths of stay (LOS) between the two cohorts.

## 2 Materials and methods

### 2.1 Study design and data source

The study was approved by the (masked for review) Institutional Review Board (IRB# 21-08375-XP). The requirement for informed consent was waived as this study involved retrospective analysis of de-identified data from the Pediatric Health Information System (PHIS), a database of pediatric clinical and resource utilization data from 45 children's hospitals (9). All procedures were conducted in accordance with institutional ethical standards and the principles of the Declaration of Helsinki. This cross-sectional study included children admitted to our institution, a single, urban Children's Hospital located in the Mid-Southern US. The hospital has 255 inpatient beds and an average of 15,441 admissions per year between 2011 and 2019. From the PHIS, the following variables were gathered: demographic data (age, sex, race); insurance type; All Patient Refined-Diagnosis Related Group (APR-DRG) severity of illness score; presence of any ICU stay during the admission; admission date and time; and discharge date and time. The APR-DRG severity score was selected as the primary severity measure because it is a standardized administrative classification used to assess inpatient illness severity, case mix, resource utilization, hospital performance, and insurance billing. At our institution, APR-DRG assignment followed the same coding and billing workflow during both the pre-pandemic and pandemic periods, with no changes in documentation, billing, or reporting practices. ICU admission and length of stay were included as additional acuity-related outcomes.

### 2.2 Study population

The study included children ≤18 years of age admitted through the hospital's emergency department to the following services: general pediatrics, cardiology, nephrology, neurology, gastrointestinal, general surgery, pulmonology, and pediatric intensive care unit (PICU). Age groups were categorized as infants younger than 1 year, preschool-aged children 1–4 years, and school-aged children 5–18 years. The school-aged category was selected intentionally to represent children and adolescents with potential school-related exposure during the pandemic period, including exposure related to school attendance, school closures, virtual learning, masking policies, reopening practices, and changes in peer interaction.

**Figure 1:**
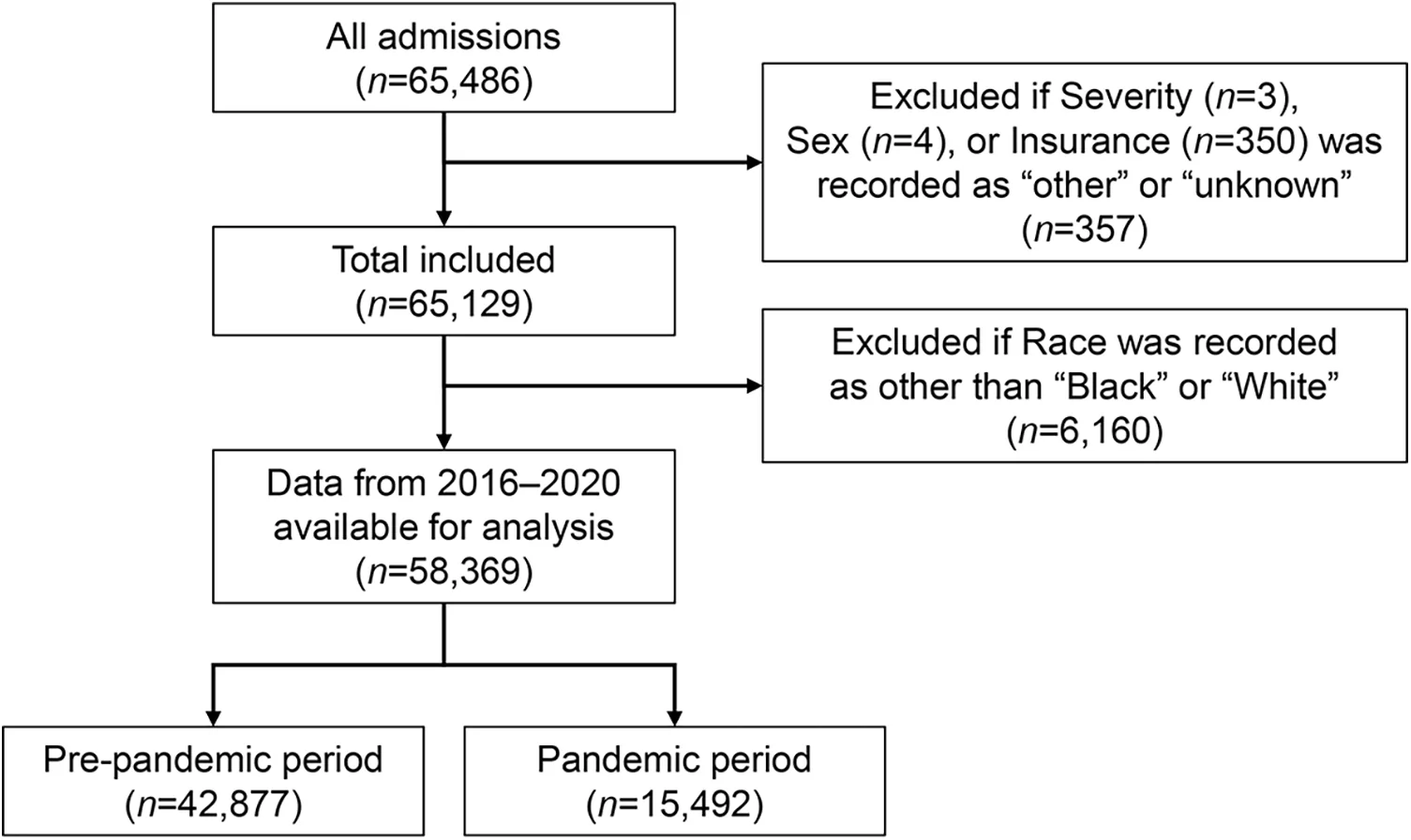
STROBE flow diagram depicting selection of admissions for analysis.

All individual admissions meeting these criteria were considered for this analysis and treated as independent events. Patients admitted to the neonatal ICU and other hospital admissions for elective procedures or diagnostic procedures were excluded. The initial dataset included 65,486 total admissions (Figure 1). Patients admitted with any admission variable described as “Unknown” or “Other” or that had any missing admission variables were excluded (*n* = 357).

Race and ethnicity categories other than Non-Hispanic Black and Non-Hispanic White were excluded (*n* = 6,760) from the primary race-based analysis because of concern for inconsistent documentation and reporting during the study period. The electronic medical record reporting method for race and ethnicity changed during the study period, which appeared to affect the distribution of Hispanic and Other categories across the pre-pandemic and pandemic periods. (Hispanic admissions increased from 176 to 690, or 4.1%, while the Other category decreased from 3,536, to 641, or 3.8%). Primary race-based analysis was thus limited to Non-Hispanic Black and Non-Hispanic White patients, the two largest and most consistently documented groups, to reduce potential misclassification bias.

### 2.3 Pre-pandemic period and pandemic period exposure

The pre-pandemic period was defined as the period when patients were admitted and discharged between January 1, 2016, and February 29, 2020, and treated as a single exposure. The pandemic period was defined as the time when patients were admitted and discharged between March 1, 2020, to December 31, 2021. Admissions before March 1, 2020, but discharged after this date were assigned to the pre-pandemic period.

### 2.4 Main outcome measures

The primary outcome measures were severity of illness (SOI), ICU admission, and LOS in days, all of which are used as indicators of illness acuity. SOI was defined using 3M APR-DRG Severity Level guidelines (10), which classifies patients according to reason for admission, severity of illness, and mortality risk (11). This system is an expansion of the basic DRGs to represent non-Medicare populations such as pediatric patients. The APR-DRG SOI is used for insurance purposes, and categorizes illnesses as Minor, Moderate, Major, and Extreme (12). These categories are commonly used by health systems to determine severity, utilization, performance, operations, and more. ICU admission indicates the incidence of any ICU admission or stay during an individual hospital stay. LOS was calculated as the elapsed time in days from the date and time of admission to the date and time of discharge.

### 2.5 Covariates

Demographic information from PHIS data included age, sex, and race. Age groups were categorized as infant (0–<1 year of age), preschool (1–4 years of age), and school age (5–18 years of age).

### 2.6 Statistical analyses

We reported mean and 95% confidence intervals (CI) for variables that approximated the normal distribution, and median and interquartile range (IQR) for skewed variables. For categorical variables, we presented counts and percentage of the various categories. Associations of continuous covariates with pandemic timing (pre-pandemic and pandemic) were reported using the Wilcoxon rank sum test. The associations of categorical covariates with pandemic timing were reported using Fisher's exact test. We then determined the unadjusted associations of categorized covariates with pandemic timing and health outcomes (the SOI and ICU admission status) using generalized linear regression models and *p*-values from the Wald test.

We subsequently evaluated the adjusted associations between the pandemic timing and the SOI and ICU admission status after controlling for age group, sex, and race. Odds ratios for the associations and *p*-values from the Wald test were reported.

The effect of the pandemic on the LOS in days was assessed using Kaplan–Meier curves for time to discharge, truncated to 30 days of duration, with the two periods compared using the log-rank test (Figure 3). Statistical significance was set as *p* < 0.05. R version 4.0.3 (R Core Team) was used for all analyses.

## 3 Results

### 3.1 Study population and demographic characteristics

**Figure 2:**
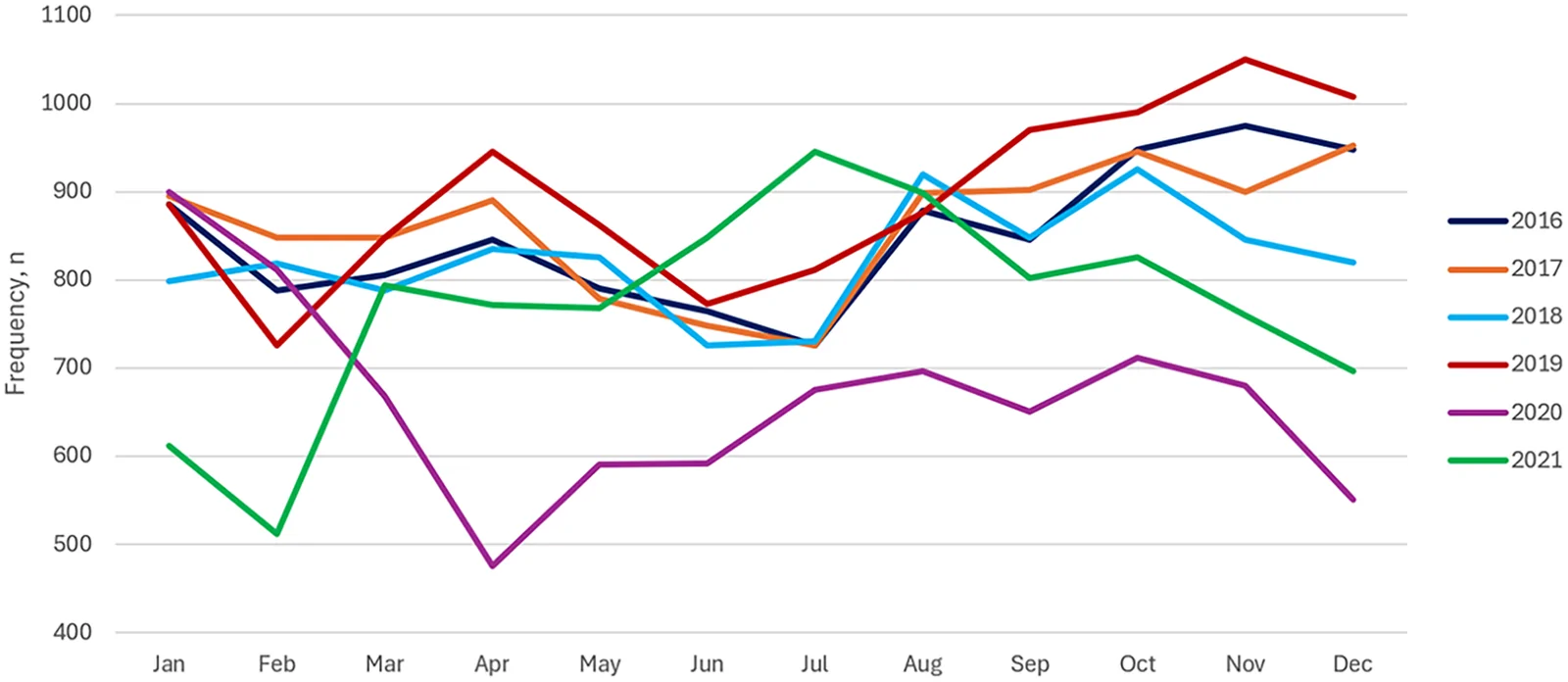
Admission trends for pandemic period compared to pre-pandemic period. Graph shows a rapid reduction of admissions starting from March 2020, with admissions remaining low for the rest of 2020 compared with pre-pandemic period. After February 2021, the rate of admissions started increasing rapidly to pre-pandemic levels.

A total of 58,369 admissions were included, with 42,877 admissions from the pre-pandemic period and 15,492 from the pandemic period (Figure 1). Admissions were relatively steady from January 2016 through February 2020, but there was a precipitous drop in monthly admissions starting mid-March and continuing through April 2020, until a rebound occurred in March 2021 (Figure 2).

Comparisons of demographic characteristics and outcome variables between pre-pandemic and pandemic periods are depicted in Table 1. The proportions of hospital admissions for the infant age group (<1 year; pre-pandemic, 22% vs. pandemic, 18%) and preschool age group (1–4 years; pre-pandemic, 27% vs. pandemic, 25%) were reduced during the pandemic. Admissions increased for the school-age group (pre-pandemic, 51% vs. pandemic, 57%; *p* ≤ .001). Our data show a slight but not clinically relevant increase in the proportion of admissions for females during the pandemic period (pre-pandemic, 46% vs. pandemic, 47%) compared to admissions for male patients whose admissions decreased slightly (*p* ≤ .001). Similarly, there was a slight and clinically irrelevant increase in the proportion of admissions among white patients (pre-pandemic, 44% vs. pandemic, 45%) compared with Black patients during in the pandemic period, whose admissions decreased slightly (*p* ≤ .012). Severity of Illness and ICU Admission Comparisons between Pre-pandemic and Pandemic Periods.

**Table 1:** Demographic characteristics and outcome variables.

Characteristic	Total (*N* = 58,369)	Pre-pandemic period, (*n* = 42,877)	Pandemic period (*n* = 15,492)	OR	95% CI
Demographics					
Age, median year (IQR)	5.0 (1.0–13.0)	5.0 (1.0–12.0)	6.0 (1.0–14.0)	–	–
Age group, *n* (%)					
Infant (<1 years)	12,110 (21%)	9,271 (22%)	2,839 (18%)	–	–
Pre-school (1–4 years)	15,409 (26%)	11,557 (27%)	3,852 (25%)	1.09	1.03–1.15
School age (5+ years)	30,850 (53%)	22,049 (51%)	8,801 (57%)	1.30	1.24–1.37
Sex, *n* (%)					
Male	31,509 (54%)	23,334 (54%)	8,175 (53%)	–	–
Female	26,860 (46%)	19,543 (46%)	7,317 (47%)	1.07	1.03–1.11
Race category, *n* (%)^a^					
Black	32,398 (56%)	23,932 (56%)	8,466 (55%)		
White	25,971 (44%)	18,945 (44%)	7,026 (45%)	1.05	1.01–1.09
Outcome variables					
Severity of illness, *n* (%)					
Minor	22,607 (39%)	17,184 (40%)	5,423 (35%)	–	–
Moderate	23,028 (39%)	17,085 (40%)	5,943 (38%)	1.10	1.06–1.15
Major	9,606 (16%)	6,608 (15%)	2,998 (19%)	1.44	1.36–1.52
Extreme	3,128 (5.4%)	2,000 (4.7%)	1,128 (7.3%)	1.79	1.65–1.93
ICU admission, *n* (%)					
No	52,406 (90%)	38,618 (90%)	13,788 (89%)	–	–
Yes	5,963 (10%)	4,259 (9.9%)	1,704 (11%)	1.12	1.06–1.19
Length of stay					
Median days (IQR)^a^	2.0 (1.0–3.0)	2.0 (1.0–3.0)	2.0 (1.0–3.0)	–	–

95% CI, 95% confidence interval; IQR, interquartile range; OR, odds ratio.

a All comparisons between the pre-pandemic and pandemic cohorts were significant (*p* < .001), except race (*p* = .012) and length of stay (*p* = .002). In the race category, all non-Black White admissions were excluded.

Proportions of admissions with various SOI designations among patients admitted based on the APR-DRG score showed that admissions for Minor Severity (pre-pandemic, 40% vs. pandemic. 35%) and Moderate Severity (pre-pandemic, 40% vs. pandemic, 38%) conditions were reduced during the pandemic period compared to the pre-pandemic period (*p* ≤ .001) (Table 1). Admissions for Major Severity (pre-pandemic, 15.4% vs. pandemic, 19.4%) and Extreme Severity (pre-pandemic, 4.7% vs. pandemic, 7.3%) conditions occurred more frequently during the pandemic period compared with the pre-pandemic period. Minor and Moderate severity levels were recategorized as “less severe,” and Major and Extreme severity levels were recategorized as “more severe.” Unadjusted and adjusted prediction models of recategorized severity level showed increased admissions of “more severe” patients during the pandemic (OR_unadjusted_ = 1.45; 95% CI, 1.38–1.51 vs. OR_adjusted_ = 1.45; 95% CI, 1.39–1.51; *p* < .001) (Table 2). Being male or White significantly increased the likelihood of a child having a “more severe” illness level, after adjusting for period (Table 2).

**Table 2:** Effect of pandemic on severity of illness and ICU admissions, unadjusted and adjusted models, OR (95% CI).

Outcome/Predictor variables	Unadjusted model	Adjusted model
Severity of Illness^a^		
Pandemic period	1.45 (1.38–1.51)	1.45 (1.39–1.51)
Age		
Pre-school (1–4 years)	–	1.00 (0.94–1.06)
School age (5+ years)	–	0.97 (0.93–1.03)
Sex		
Female	–	0.91 (0.88–0.94)
Race		
White	–	1.05 (1.01–1.09)
ICU admission^a^		
Pandemic period	1.12 (1.06–1.19)	1.14 (1.07–1.21)
Age		
Pre-school (1–4 years)	–	0.84 (0.78–0.90)
School age (5+ years)	–	0.88 (0.73–0.84)
Sex		
Female	–	0.88 (0.83–0.93)
Race		
White	–	0.80 (0.76–0.84)

95% CI, 95% confidence interval; ICU, intensive care unit; OR, odds ratio.

a Outcome variables.

The proportion of admissions to the ICU increased during the pandemic period (pre-pandemic, 9.9% vs. pandemic, 11.0%) compared with the pre-pandemic period (*p* ≤ .001) (Table 1). Unadjusted and adjusted prediction models of ICU admission showed increased admission rates during the pandemic (OR_unadjusted_ = 1.12; 95% CI, 1.06–1.19 vs. OR_adjusted_ = 1.14; 95% CI, 1.07–1.21; *p* < .001) (Table 2). Being between 5 and 18 years of age (i.e., the school-age category), male, or Black significantly increased the likelihood of a child's admission to the ICU after adjusting for period (Table 2).

### 3.2 Length of stay comparisons between pre-pandemic and pandemic periods

**Figure 3:**
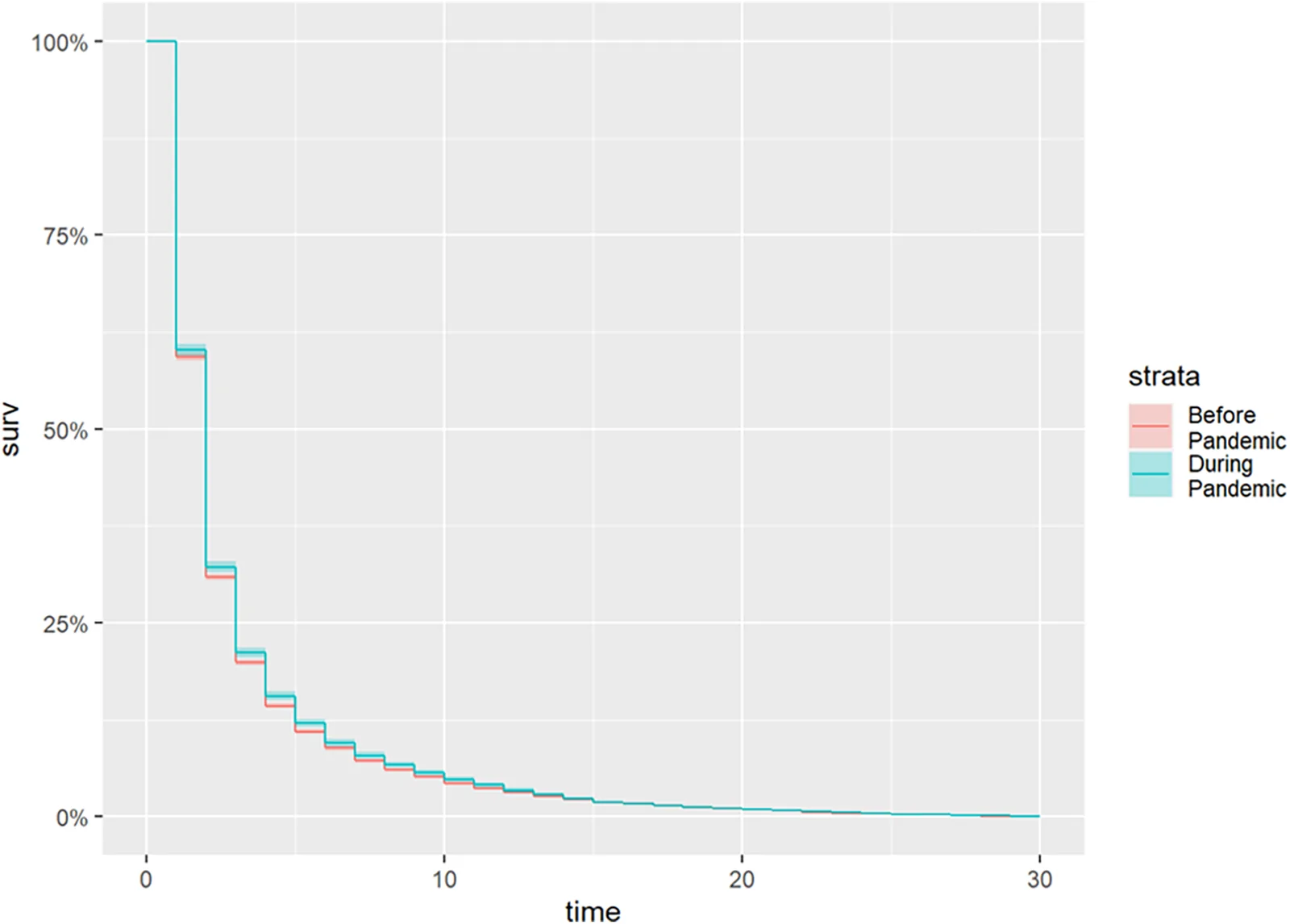
Kaplan–Meier curves for length of stay comparing time to discharge from hospital for during the pandemic and pre-pandemic periods (*X* axis, time in days; *Y* axis, survival percentage).

The median LOS was 2 days during both the pre-pandemic and pandemic periods (*p* < .002). While this is a statistically significant finding, this difference is attributable to fractions of days (i.e., a few hours) and is clearly not clinically meaningful. Comparison of the LOS over 30 days during the pandemic and pre-pandemic periods showed that the difference in LOS between two periods was small but consistent 15 days before (*p* = .008) (Figure 3).

## 4 Discussion

The primary objective of this study was to compare overall pediatric hospitalization patterns during pre-pandemic and pandemic periods, The pandemic period was analyzed as a single exposure period from March 1, 2020, through December 31, 2021. Although phase-specific subgroup analyses were not performed, monthly admission trends are presented in Figure 2, which demonstrates the marked decline in admissions beginning in March 2020, continued lower admission volume during much of 2020, and the subsequent increase in admissions toward pre-pandemic period levels in 2021.

To our knowledge, this study is one of the few in the literature designed to measure the severity level of pediatric patients admitted to the hospital during the COVID-19 pandemic compared with pre-pandemic norms using the APR-DRG severity score. This study found an increased proportion of admissions labeled as “Major” and “Extreme” compared with admissions labeled as “Minor” and “Moderate” during the pandemic period; pandemic period admissions also increased compared with pre-pandemic admissions. One reason for this increase may be attributable to caregivers' avoidance of healthcare facilities during the pandemic. Such avoidance could have caused delays in presentation for evaluation and care by healthcare personnel, loss of routine care and preventive care opportunities, loss of chronic care management visits, or postponement of elective surgical procedures, thus leading to more severe presentations on admission (13–15). During the pandemic, there was also reduced access to school-based nutritional and health services, which may have resulted in poorer health and less opportunities for trained professionals to evaluate children at the onset of illness.

Another possible explanation is that parents were working from home or experiencing joblessness, while their children attended school virtually. These situations may have allowed caregivers more opportunities to “watch and wait” an illness or injury without worrying about missing school days or work. They might have sought earlier evaluations to quickly return to school or work. The virtual school environment during the pandemic also caused a drop in demand for doctor's visits, due in part to the lack of reporting by teachers in the school environment on students missing multiple school days, poor mental health, or signs of physical abuse, to name a few examples. Additionally, parents who lost their jobs may have also lost their health insurance coverage.

A recent cross-sectional study showed that COVID-19 disproportionately affected low-income households and racial and ethnic minority communities (16). That study found that low socioeconomic status populations and Black and multiracial families had poor continuity of care to well-child visits and follow-up appointments for chronic medical condition during the pandemic compared with the White population. Many adults and children experienced poor mental health throughout the pandemic, including depression and anxiety, which could have led to avoidance of the healthcare system or loss of motivation or agency, while also leading to more instances of abuse, trauma, or self-harm behaviors. Patients with these types of negative behaviors may present with higher acuity requiring higher levels of care.

Baseline comorbidity burden may have contributed to the observed increase in severity of illness and ICU admission during the pandemic period. Children who required hospitalization during the pandemic period may have represented a different case mix than children admitted during the pre-pandemic period. Disruptions in routine care, chronic disease management, outpatient follow-up, school-based services, and caregiver access to healthcare in particular may have disproportionately affected children with chronic medical conditions or medical complexity. Patients with technology dependence, immunocompromised status, obesity, asthma, neurologic disorders, or other chronic conditions may have been more vulnerable to delayed care and more severe presentation. Therefore, the observed increase in APR-DRG SOI and ICU admission should be interpreted in the context of potential differences in baseline medical complexity and hospitalized patient case mix.

Finally, to avoid exposure to the SARS-CoV-2 virus, many sheltered in place and avoided medical clinics, emergency rooms, and hospitals, which were potential sources of high exposure. It is likely that a combination of these circumstances led to the findings in our study.

The U.S. Centers for Disease Control (CDC) reported that even though mortality and hospital admissions during the pandemic were low in the pediatric populations, the presence of underlying medical conditions were found to be a risk factor for children requiring hospitalization or ICU admission for COVID-19 (17). Our data showed a significant increase in ICU admissions during the pandemic period (pre-pandemic period, 9.9% vs. pandemic period, 11%; *p* < .001), which supports the finding of increased levels of SOI during the pandemic period. The reason for increased ICU admissions could be multifactorial. One reason for pediatric pandemic ICU admissions was Multisystem Inflammatory Syndrome in Children (MIS-C). As of October 2021, there were 196 cases of MIS-C in Tennessee, and 45% of those cases were admitted to the ICU (18). A previous study on MIS-C showed most patients affected were around 6–12 years old, with 73% of that group admitted to the ICU (19). Another reason for the significant increase in ICU admission during the pandemic period could be that patients with underlying conditions received little or no follow-up by medical personnel, practiced social distancing, and feared exposure to COVID-19 in medical settings.

A previous study reported that increased psychological stressors among both children and their caregivers due to social isolation resulted in increased incidences of child abuse, intentional ingestion, and suicide-related admissions, all of which often require ICU level of care (20). A study analyzing PICU admission diagnoses comparing pre-pandemic and pandemic periods reported a significant reduction in PICU admissions due to respiratory causes but increased admissions for diabetes, poisoning/ingestion, cancer, and neurovascular disease (17).

Considering the various age groups, our study showed school-aged children (5–18 years) had higher levels of admissions during the pandemic period (pre-pandemic period, 51% vs. pandemic period, 57%; OR = 1.30; 95% CI, 1.24–1.37; *p* < 0.001) compared with infants (pre-pandemic period, 22% vs. pandemic period, 18%) and preschool aged children (1–4 years) (pre-pandemic period, 27% vs. pandemic period, 25%) (Table 1). CDC data showed a doubling of the weekly incidence of COVID-19 diagnoses in adolescents (12–17 years of age) compared with children aged 5–11 years (21) in the early stages of the pandemic period. Our findings of increased admission in school-age children align with these CDC data showing increased admissions in the adolescent age group during the pandemic period.

The findings for LOS outcomes were statistically significant but likely without practical significance. One previously reported study showed that disease severity was lower for respiratory and neurological disease throughout the pandemic, but LOS for gastrointestinal disease admissions was higher (22). This result might be attributable to delayed presentation resulting in an increase in the severity of illness. Another study of inpatient admissions for a children's hospital during the early pandemic showed an overall decrease in LOS (23).

### 4.1 Limitations

Limitations associated with this study include using APR-DRG SOI score is an administrative measure used for severity classification and billing purposes, which may not capture all clinical indicators of severe illness, such as mechanical ventilation, vasopressor use, ECMO support, mortality, or PICU length of stay. These variables were beyond the scope of the original study design and were not included in the primary analytic dataset. Additionally, although APR-DRG SOI partially accounts for patient complexity through diagnosis, complication, and comorbidity coding, this study did not incorporate a separate pediatric comorbidity index nor did it directly adjust for individual chronic conditions, such as technology dependence, immunocompromised status, obesity, asthma, or neurologic disorders. Therefore, residual confounding by baseline comorbidity burden may remain. The findings should be interpreted as differences in APR-DRG administrative SOI and ICU admission during the pandemic period, rather than definitive evidence of increased clinical severity independent of comorbidity burden.

Another limitation is the exclusion of Asian, Hispanic, or Other from the primary race-based analysis because race and ethnicity documentation changed during the study period. Although this approach was used to reduce potential misclassification bias, it may have introduced selection bias and may limit the generalizability of race-based findings.

The school-aged category included children and adolescents aged 5–18 years. Although this grouping was selected to reflect potential school-related exposure during the pandemic, it includes children at different developmental stages. Disease patterns, injury risk, behavioral health concerns, substance use, and healthcare utilization may differ between younger school-aged children and adolescents. Therefore, this classification may obscure age-specific differences within the school-aged population. Future studies could further stratify school-aged children into younger children and adolescents to evaluate whether pandemic-associated hospitalization patterns differed across developmental subgroups.

Finally, this study analyzed the pandemic period as a single exposure period and did not stratify by specific pandemic phases, such as the early pandemic period, winter respiratory infection waves, Delta/Omicron waves, or pre- and post-vaccination pediatric periods. Therefore, the analysis may not capture phase-specific differences in severity of illness, ICU admission, or length of stay. Future studies should evaluate these outcomes across specific pandemic phases.

## 5 Conclusion

This study not only showed an overall reduction of hospital admissions in pediatric age groups during the initial phase of pandemic period, but that hospitalized pediatric patients demonstrated higher APR-DRG administrative SOI scores and higher ICU admission rates in the pandemic period, also. These findings may reflect delayed care, altered admission patterns, changes in hospitalized patient case mix, and differences in baseline medical complexity. While there were statistically significant differences in LOS, they were unlikely to be clinically significant. These findings indicated that children who did require admission during the pandemic period were more severely ill and required higher levels of care. This finding reinforces concerns that children missed opportunities for early evaluation and care due to the measures taken to reduce viral spread. It also reinforces the need for systems of care for pediatric patients even during a global pandemic. While these infection prevention measures saved lives and prevented hospital admissions as well, routine care and chronic disease management for children must be prioritized and balanced with the need to reduce infections.

## Data Availability

The original contributions presented in the study are included in the article/Supplementary Material, further inquiries can be directed to the corresponding author.

## References

[1] Cucinotta D Vanelli M. WHO declares COVID-19 a pandemic. Acta Biomed. (2020) 91(1):157–60. 10.23750/abm.v91i1.9397 32191675 PMC7569573

[2] Ren X. Pandemic and lockdown: a territorial approach to COVID-19 in China, Italy and the United States. Eurasian Geogr Econ. (2020) 61(4–5):423–34. 10.1080/15387216.2020.1762103

[3] Today USA. COVID-19 restrictions: map of COVID-19 case trends and restrictions: USA Today (2022). Available online at: https://www.usatoday.com/storytelling/coronavirus-reopening-america-map/ (Accessed May 9, 2022).

[4] Mann E Swedien D Hansen J Peterson S Saheed M Klein E. Reduction in emergency department presentations in a regional health system during the COVID-19 pandemic. West J Emerg Med. (2021) 22(4):842–50. 10.5811/westjem.2020.10.49759 35354014 PMC8328168

[5] DeLaroche AM Rodean J Aronson PL Fleegler EW Florin TA Goyal M. Pediatric emergency department visits at US children’s hospitals during the COVID-19 pandemic. Pediatrics. (2021) 147(4):1–11. 10.1542/peds.2020-039628 33361360

[6] Potts AL Thomas A Bork SJD Cutting P Nguyen TT Tracy J. Safely caring for adult patients in a pediatric hospital during the COVID-19 pandemic: a focus on the medication-use process. Am J Health Syst Pharm. (2021) 78(12):1128–33. 10.1093/ajhp/zxab074 33634315 PMC7989610

[7] Samieefar N Rashedi R Akhlaghdoust M Mashhadi M Darzi P Rezaei N. Delta variant: the new challenge of COVID-19 pandemic, an overview of epidemiological, clinical, and immune characteristics. Acta Biomed. (2022) 93(1):e2022179. 10.23750/abm.v93i1.12210 35315394 PMC8972886

[8] Kumar S Thambiraja TS Karuppanan K Subramaniam G. Omicron and delta variant of SARS-CoV-2: a comparative computational study of spike protein. J Med Virol. (2022) 94(4):1641–9. 10.1002/jmv.27526 34914115

[9] Olson D Birkholz M Gaensbauer JT Asturias EJ Todd JK. Analysis of the pediatric health information system database as a surveillance tool for travel-associated infectious diseases. Am J Trop Med Hyg. (2015) 92(5):1067–9. 10.4269/ajtmh.14-0794 25778506 PMC4426564

[10] US Centers for Medicare and Medicaid Services. ICD-10-CM/PCS MS-DRG v35 definitions manual table of contents - full titles - HTML versions (2017) Baltimore, MD: US Centers for Medicare & Medicaid Services (2017). Available online at: https://www.cms.gov/icd10m/version35-fullcode-cms/fullcode_cms/P0001.html (Accessed March 17, 2026).

[11] Horn SD Horn RA Sharkey PD. The severity of illness index as a severity adjustment to diagnosis-related groups. Health Care Financ Rev. (1984) 33–45. 10311075 PMC4195109

[12] Averill RF Goldfield N Hughes JS Bonazelli J McCullough EC Steinbeck BA. All patient refined diagnosis related groups (APR-DRGS): methodology overview (version 20.0) [PDF]. 3M Health Information Systems (2003). Available online at: https://hcup-us.ahrq.gov/db/nation/nis/APR-DRGsV20MethodologyOverviewandBibliography.pdf (Accessed March 17, 2026).

[13] Ciacchini B Tonioli F Marciano C Faticato MG Borali E Pini Prato A. Reluctance to seek pediatric care during the COVID-19 pandemic and the risks of delayed diagnosis. Ital J Pediatr. (2020) 46(1):87. 10.1186/s13052-020-00849-w 32600464 PMC7322712

[14] Araujo OR Almeida CG Lima-Setta F Prata-Barbosa A Colleti Junior J, Brazilian Research Network in Pediatric Intensive Care. The impact of the novel coronavirus on Brazilian PICUs. Pediatr Crit Care Med. (2020) 21(12):1059–63. 10.1097/PCC.0000000000002583 32925566 PMC7508037

[15] Meade J. Mental health effects of the COVID-19 pandemic on children and adolescents: a review of the current research. Pediatr Clin North Am. (2021) 68(5):945–59. 10.1016/j.pcl.2021.05.003 34538305 PMC8445752

[16] Batioja K Elenwo C Hartwell M. Disparities in pediatric medical and childcare disruption due to COVID-19. JAMA Pediatr. (2023) 177(4):432–4. 10.1001/jamapediatrics.2022.6130 36806461 PMC9941966

[17] Zee-Cheng JE McCluskey CK Klein MJ Scanlon MC Rotta AT Shein SL. Changes in pediatric ICU utilization and clinical trends during the coronavirus pandemic. Chest. (2021) 160(2):529–37. 10.1016/j.chest.2021.03.004 33727033 PMC7954775

[18] Torres JP Izquierdo G Acuna M Pavez D Reyes F Fritis A. Multisystem inflammatory syndrome in children (MIS-C): report of the clinical and epidemiological characteristics of cases in Santiago de Chile during the SARS-CoV-2 pandemic. Int J Infect Dis. (2020) 100:75–81. 10.1016/j.ijid.2020.08.062 32861823 PMC7452906

[19] Feldstein LR Tenforde MW Friedman KG Newhams M Rose EB Dapul H. Characteristics and outcomes of US children and adolescents with multisystem inflammatory syndrome in children (MIS-C) compared with severe acute COVID-19. J Am Med Assoc. (2021) 325(11):1074–87. 10.1001/jama.2021.2091 PMC7905703 33625505

[20] Kaplan LJ Kleinpell R Maves RC Doersam JK Raman R Ferraro DM. Critical care clinician reports on coronavirus disease 2019: results from a national survey of 4,875 ICU providers. Crit Care Explor. (2020) 2(5):e0125. 10.1097/CCE.0000000000000125 32671350 PMC7259564

[21] Leeb RT Price S Sliwa S Kimball A Szucs L Caruso E. COVID-19 trends among school-aged children - United States, March 1-September 19, 2020. Morb Mortal Wkly Rep. (2020) 69(39):1410–5. 10.15585/mmwr.mm6939e2 PMC7537558 33001869

[22] Bogli J Gusewell S Strassle R Kahlert CR Albrich WC. Pediatric hospital admissions, case severity, and length of hospital stay during the first 18 months of the COVID-19 pandemic in a tertiary children’s hospital in Switzerland. Infection. (2023) 51(2):439–46. 10.1007/s15010-022-01911-x 36065045 PMC9444086

[23] Markham JL Richardson T DePorre A Teufel RJ 2nd Hersh AL Fleegler EW. Inpatient use and outcomes at children’s hospitals during the early COVID-19 pandemic. Pediatrics. (2021) 147(6):1–9. 10.1542/peds.2020-044735 33757994

